# Health Facility Characteristics and Their Relationship to Coverage of PMTCT of HIV Services across Four African Countries: The PEARL Study

**DOI:** 10.1371/journal.pone.0029823

**Published:** 2012-01-20

**Authors:** Didier K. Ekouevi, Elizabeth Stringer, David Coetzee, Pius Tih, Tracy Creek, Kathryn Stinson, Andrew O. Westfall, Thomas Welty, Namwinga Chintu, Benjamin H. Chi, Cathy Wilfert, Nathan Shaffer, Jeff Stringer, Francois Dabis

**Affiliations:** 1 Programme PAC-CI, Abidjan, Cote d'Ivoire, France; 2 Institute National de la Santé et de la Recherche Médicale, Université Victor Segalen, Bordeaux, France; 3 Centre for Infectious Disease Research in Zambia, Lusaka, Zambia; 4 University of Cape Town, Cape Town, South Africa; 5 Cameroon Baptist Health Convention Health Board, Bamenda, Cameroon; 6 United States Centers for Disease Control and Prevention, Atlanta, Georgia, United States; 7 Elizabeth Glaser Pediatric AIDS Foundation, Washington, D.C., United States of America; 8 World Health Organization, Geneva, Switzerland; University of California, San Francisco, United States of America

## Abstract

**Background:**

Health facility characteristics associated with effective prevention of mother-to-child transmission of HIV (PMTCT) coverage in sub-Saharan are poorly understood.

**Methodology/Principal Findings:**

We conducted surveys in health facilities with active PMTCT services in Cameroon, Cote d'Ivoire, South Africa, and Zambia. Data was compiled via direct observation and exit interviews. We constructed composite scores to describe provision of PMTCT services across seven topical areas: antenatal quality, PMTCT quality, supplies available, patient satisfaction, patient understanding of medication, and infrastructure quality. Pearson correlations and Generalized Estimating Equations (GEE) to account for clustering of facilities within countries were used to evaluate the relationship between the composite scores, total time of visit and select individual variables with PMTCT coverage among women delivering.

Between July 2008 and May 2009, we collected data from 32 facilities; 78% were managed by the government health system. An opt-out approach for HIV testing was used in 100% of facilities in Zambia, 63% in Cameroon, and none in Côte d'Ivoire or South Africa. Using Pearson correlations, PMTCT coverage (median of 55%, (IQR: 33–68) was correlated with PMTCT quality score (rho = 0.51; p = 0.003); infrastructure quality score (rho = 0.43; p = 0.017); time spent at clinic (rho = 0.47; p = 0.013); patient understanding of medications score (rho = 0.51; p = 0.006); and patient satisfaction quality score (rho = 0.38; p = 0.031). PMTCT coverage was marginally correlated with the antenatal quality score (rho = 0.304; p = 0.091). Using GEE adjustment for clustering, the, antenatal quality score became more strongly associated with PMTCT coverage (p<0.001) and the PMTCT quality score and patient understanding of medications remained marginally significant.

**Conclusions/Results:**

We observed a positive relationship between an antenatal quality score and PMTCT coverage but did not identify a consistent set of variables that predicted PMTCT coverage.

## Introduction

Despite the universal recognition of efficacious interventions and unprecedented resources to reduce MTCT (mother to child transmission), in 2009, only 26% of pregnant women living in low and middle income countries had been tested for HIV and among identified HIV-positive women, only an estimated 53% received an antiretroviral (ARV) regimen to prevent mother-to-child HIV transmission (PMTCT) [1]. As a result of the failure of health systems to effectively deliver PMTCT regimens, approximately 1,000 children under the age of 15 continue to be infected with HIV every day, the vast majority around the time of birth and through breastfeeding [1].

While multiple studies have measured PMTCT efficacy, studies that describe determinants of PMTCT program effectiveness are rare. The few published reports that have assessed PMTCT program effectiveness have not addressed reasons for successes and failures [2]–[4]. A multi-site PMTCT effectiveness study in four African countries called PMTCT Effectiveness in Africa: Research and Linkages to Care (the PEARL Study) was conducted between 2007–2009, and found that coverage of nevirapine (NVP) among HIV-infected women delivering in health facilities with PMTCT services varied dramatically and was only 55% overall [5].

A better understanding of barriers to high PMTCT coverage is needed with the new 2010 World Health Organization (WHO) guidelines promoting more efficacious ARV combination regimens for women and extended infant prophylaxis during breastfeeding. Implementation of the new guidelines will require higher quality antenatal - and PMTCT care, more rigorous infant follow up, and ongoing maternal care after delivery [6]. More complex PMTCT regimens will also require a greater understanding of what occurs at the health facility level and where investments need to be made [1], [7], [8]. Using data from the PEARL Study, we examine how facility and service characteristics predict maternal-infant coverage with NVP at the time of delivery.

## Methods

The PEARL study was a multi-country evaluation of PMTCT effectiveness at the patient, facility, and community levels. We previously reported PMTCT coverage in a survey among women delivering in 43 health facilities in Cameroon, Cote d'Ivoire, South Africa, and Zambia between 2007 and 2009 [5]. PMTCT coverage was defined as the proportion of HIV-positive mother-HIV-exposed baby pairs in which both received single-dose NVP. Maternal dosing was confirmed by biochemical measurement in the cord blood and newborn dosing was confirmed by chart review [5]. As part of this study, we completed facility surveys at delivery centers which also provided antenatal care.

### Data collection

We used a modified version of “A Rapid Health Facility Assessment Tool: to Enhance Quality and Access at the Primary Health Care Level.” This tool was developed in 2006 by ICF Macro in collaboration with MEASURE Evaluation and a panel of experts from the United States Agency for International Development (USAID) and other cooperating agencies and modified to include detailed PMTCT information in parallel with other antenatal care information. The original tool is available online http://www.mchipngo.net/controllers/link.cfc?method=tools_rhfa.

The questionnaire included general questions such as type of facility, estimated size of the catchment area, and location as well as four discrete modules on clinic operations. Each module contained 32–120 questions. Modules included direct observation of clinician patient encounters, exit interviews of patients, and questions of patients and providers.

- Module 1 was a quality-of-care checklist completed by direct observation of an antenatal consultation. Up to six consecutive antenatal visits in each facility were observed. Only first ANC visit observations were used.
- Module 2 was an exit interview with up to six consecutive pregnant women after the consultation to measure patient perceptions about care. Key measurements were satisfaction and understanding of each medicine or prescription given during the consultation.
- Module 3 was a checklist of available infrastructure, equipment and supplies (including drugs).
- Module 4 was an interview with the health center manager regarding services provided, numbers of personnel, and other staffing variables.

The survey instrument was adapted and translated into French in Côte d'Ivoire and was pretested in the four countries prior to data collection. All questionnaires were entered into a Microsoft Access database and sent to PEARL's central data management and analysis unit.

We examined each variable (n = 377) in the questionnaire individually to determine which ones were associated with PMTCT coverage. In addition, we created seven composite scores that summarized features of the clinic in several domains in a systematic manner. These scores were developed *a priori* by the study co-investigators based on logical groupings of characteristics, and included composite scores for antenatal care, PMTCT, supplies, staffing level, patient satisfaction, general infrastructure, and patient understanding of medications. Scores were adjusted by country to account for different standards of care (e.g. items relating to malaria were not considered for South Africa since South Africa does not include malaria prophylaxis in routine antenatal care). Table S1 summarize the variables used and how the scores were constructed.

In addition, time-motion variables were constructed from the average of up to six patient observations at each facility. Time was recorded at a) registration b) start of exam and c) visit finish (including receipt of medication at pharmacy), allowing the calculation for the median total time of the visit as well as the time spent post test counseling.

Antenatal and PMTCT domains were assigned a score of (1) if appropriate care or treatment was given; (0.5) if appropriate care or treatment was given but not recorded in the chart; and (0) if appropriate care was not given. This was done because failure to record information, such as an HIV test, CD4 count, or blood pressure reading will lead to failure to act upon this critical information throughout the pregnancy. Infrastructure, supplies, and staff domains were assigned a score of (1) when available and (0) when not available. Scores were totaled for each domain, and divided by the number of items included. Non-applicable information and missing items were not considered in the scoring. Final domain scores were thus a proportion between 0 and 1, with a higher score reflecting more appropriate care. Patient satisfaction was scored on a scale of 1 to 5, with 5 being extremely satisfied and 1 being extremely unsatisfied. For patient comprehension, (1) point was given for each correct response for each criteria (dose, frequency, duration, and purpose), and (0) points were given for incorrect responses. (Table 1 summarizes the construction of health facility quality scores.

**Table 1:** Health facility characteristics by country; PEARL Facility Survey, 2007–2009.

	Cameroon(n = 8)	Cote d'Ivoire(n = 9)	South Africa*(n = 6)	Zambia(n = 9)	Overall(N = 32)
**Type of facility (%)**					
Hospital	63%	22%	0%	33%	31%
Health Center	38%	78%	100%	67%	69%
**Managing authority (%)**					
Government/Public	50%	89%	100%	78%	78%
NGO, Faith-based, Private	50%	11%	0%	22%	22%
**Urban/Rural (%)**					
Rural	63%	33%	67%	56%	53%
Urban	38%	67%	33%	44%	47%
**Education talk** £ **(%)**					
No	0%	11%	0%	0%	3%
Yes	100%	89%	100%	100%	97%
**Total catchment population** – median (IQR): inhabitants	38,177 (19,622–42,135)	96,597 (58,216–169,320)	26,229 (12,000–37,729)	20,841 (17,113–29,871)	38,089(20,232–70,910)
**Under five children in catchment area** - median (IQR)	4,556 (1,527–7,585)	10,124 (4,845–19,536)	500 (481–3,118)	2,408 (1,678–6,278)	3,118 (1,326–9,126)
**HIV pre-test counseling** (%)					
Group	38%	33%	0%	100%	47%
Individual	38%	67%	100%	0%	47%
Not Done	25%	0%	0%	0%	6%
**HIV testing (%)**					
Opt In	38%	100%	100%	0%	56%
Opt-Out	63%	0%	0%	100%	44%
**CD4 testing available (%)**					
All or most days	25%	33%	100%	44%	47%
No	75%	67%	0%	56%	53%
**ART available for pregnant women (%)**					
Provided at this clinic	38%	89%	33%	78%	63%
Transfer to other clinics	63%	11%	67%	22%	38%

£ : **Note that this item is not related to HIV, but to maternal and infant health in general.**

*Survey was conducted in delivery facilities where antenatal activities are implemented.

IQR: Interquartile range.

### Statistical analysis

All PEARL study facilities with cord blood and facility survey data were included. Site characteristics were summarized using descriptive statistics. We computed means and standard deviations or medians and inter-quartile ranges for continuous variables and percentages for categorical variables. Using all facilities from all 4 countries and PMTCT coverage as a continuous outcome measure, separate regression models were fit for health facility characteristics of interest and the quality scores. Generalized estimating equations (GEE) with an exchangeable correlation structure were used to account for country program -related correlation between facilities in the same country.

Statistical analyses were performed using SAS® version 9.1.3 (SAS Institute, Cary, NC, USA) and Stata® version 10.0 (StataCorp. 2007. Stata Statistical Software: Release 10. College Station, TX).

### Ethical review and role of the funding source

Approval was provided by institutional or national review boards at the U.S. Centers for Disease Control and Prevention (CDC), the University of Alabama at Birmingham, and each of the participating countries a) Comite d'éthique des sciences et de la vie, Ministère de la Santé, Côte d'Ivoire, b) University of Zambia Research Ethics Committee, Lusaka Zambia, c) Cameroon Baptist Convention Health Board, Bamenda, Northwest Province, Cameroon, d) South African Medical Research Council Ethics Committee, Cape Town, South Africa.

Verbal consent was obtained among the health care workers and the selected pregnant women who participated in the direct interview during this survey. The corresponding author had access to all data in the study and final responsibility for the decision to submit this manuscript.

## Results

### Description of the health facilities

Cord blood and facility survey data were collected from 32 facilities (8 facilities in Cameroon, 9 facilities in Cote d'Ivoire, 6 in South Africa, and 9 in Zambia). Most were health centers managed by their respective governments. General characteristics of facilities and their services are described in Table 1, An opt-out approach for HIV testing was used in 100% of facilities in Zambia, 63% in Cameroon, and none in Côte d'Ivoire and South Africa (Table 1) Antenatal care service provision among observed patients by country is described in Table 2.

**Table 2:** Antenatal care service provision among observed patients, by country; PEARL Facility Survey, 2007–2009†.

	Cameroon(n = 8)	Cote d'Ivoire(n = 9)	South Africa(n = 6)	Zambia(n = 9)	Overall(N = 32)
**Patient weighed** - mean (std)	100 (0)	100 (0)	100 (0)	100 (0)	100 (0)
**Blood pressure taken** - mean (std)	90 (19)	80 (40)	100 (0)	98 (6)	92 (24)
**Fundal height palpated** - mean (std)	98 (6)	89 (33)	100 (0)	98 (6)	96 (18)
**Fetal heart tones checked**- mean (std)	83 (37)	56 (43)	100 (0)	98 (6)	83 (33)
**Urine dipped for protein** – mean (std)	57 (53)	73 (41)	100 (0)	0 (0)	53 (49)
**Blood drawn for syphilis test** - mean (std)	54 (13)	18 (27)	100 (0)	87 (33)	62 (40)
**Blood drawn for hemoglobin test** - mean (std)	64 (24)	12 (24)	100 (0)	78 (37)	60 (42)
**Blood drawn for HIV test** – mean (std)	71 (27)	70 (38)	97 (7)	100 (0)	84 (27)
**Number of CBS mother/baby pairs** - median (IQR)	81(67–122)	52(34–84)	22(21–115)	71(54–142)	67(33–120)
**Full coverage** ** - median (IQR)	78 (59–84)	15 (9–18)	56 (48–59)	59 (54–62)	55 (33–68)

CBS: Cord blood specimens, IQR: Interquartile range, std: standard deviation.

**full coverage defined as the proportion of HIV-exposed infants in the sample with both maternal nevirapine ingestion (confirmed by cord blood chromatography) and infant nevirapine ingestion (confirmed by direct observation).

† For the direct observation patient measures we first calculated the percent observed at each facility among the up to 6 cases and then computed the mean (and standard deviation) of those facility-level percents to get the overall country values shown in the table.

### Relationship of individual facility and service characteristics to NVP coverage

Table S2 shows the relationships between facility characteristics, observed service provision, and NVP coverage. Higher coverage was associated with HIV test kits being found in ANC (as opposed to in the lab), HIV testing available in labor ward, availability of CD4 testing at the clinic, infant testing with PCR available most days of the month, and the presence of an antenatal register with PMTCT information. PMTCT coverage was not associated with type of facility, location of HIV testing during antenatal care, reported opt-in or opt-out testing strategy, same day HIV results, partner HIV testing, time of dispensation of NVP (at first antenatal visit, later in pregnancy or upon arrival at the facility in labor), or the presence of lay counselors.

### Relationship of a priori composite quality scores to NVP coverage

Table 3 shows the seven composite quality scores and two time variables stratified by country, and their relationship with NVP coverage. In univariate analysis using Pearson correlation (not adjusted for the clustering of facilities within countries), PMTCT coverage was correlated with higher PMTCT quality score (p = 0.003); infrastructure quality score (p = 0.017); patient satisfaction quality score (p = 0.031); and patient understanding of medications score (p = 0.006), and was marginally correlated with the antenatal quality score (p = 0.091). There was no correlation between PMTCT coverage and staff quality score (p = 0.168) or supply quality score (p = 0.812). PMTCT coverage was associated with longer total time at the clinic (p = 0.013), but not longer time spent for post-test counseling (p = 0.938).

**Table 3:** Health facility quality scores and correlation with PMTCT coverage, PEARL Facility Survey, 2007–2009.

	Cameroon	Cote d'Ivoire	South Africa	Zambia	Overall	ρ	p value	GEE estimate	GEE p-value*
ANC quality score – median (IQR)	67 (58–76)	62 (61–69)	77 (60–81)	67 (64–73)	67 (61–74)	0.304	0.091	4.81	<0.001
PMTCT quality score - median (IQR)	72 (67–78)	64 (60–68)	86 (78–88)	74 (70–80)	74 (67–81)	0.509	0.003	5.89	0.071
Staff quality score – median (IQR)	66 (62–73)	62 (56–75)	69 (60–76)	74 (72–82)	72 (60–78)	0.250	0.168	2.60	0.243
Supplies quality score - median (IQR)	76 (68–83)	73 (68–78)	51 (46–54)	68 (62–90)	70 (58–80)	−0.043	0.812	−0.62	0.340
Infrastructure quality score - median (IQR)	87 (67–92)	69 (62–75)	79 (75–81)	65 (65–81)	75 (65–82)	0.425	0.017	2.44	0.088
Patient satisfaction quality score - median (IQR)	4 (4-4)	4 (4-4)	4 (4–5)	4 (4-4)	4 (4-4)	0.383	0.031	5.78	0.060
Patient understanding of medications score - median (IQR)	83 (74–89)	50 (44–81)	86 (72–100)	99 (94–100)	86 (69–97)	0.507	0.006	0.02	0.986
Total time (minutes)	281(212–325)	154(113–239)	268(243–293)	327(230–379)	250 (160–327)	0.472	0.013	0.36	0.743
Post-test counseling time (minutes)	4 (3–4)	5 (3–6)	4 (2–5)	13 (7–14)	5 (4–9)	0.016	0.938	0.22	0.145

ρ: Pearson correlation coefficient, IQR: Interquartile range.

*p-value for univariate regression models treating coverage as a continuous measure, using GEE to account for correlation due to clustering of facilities within countries.

**Figure 1:**
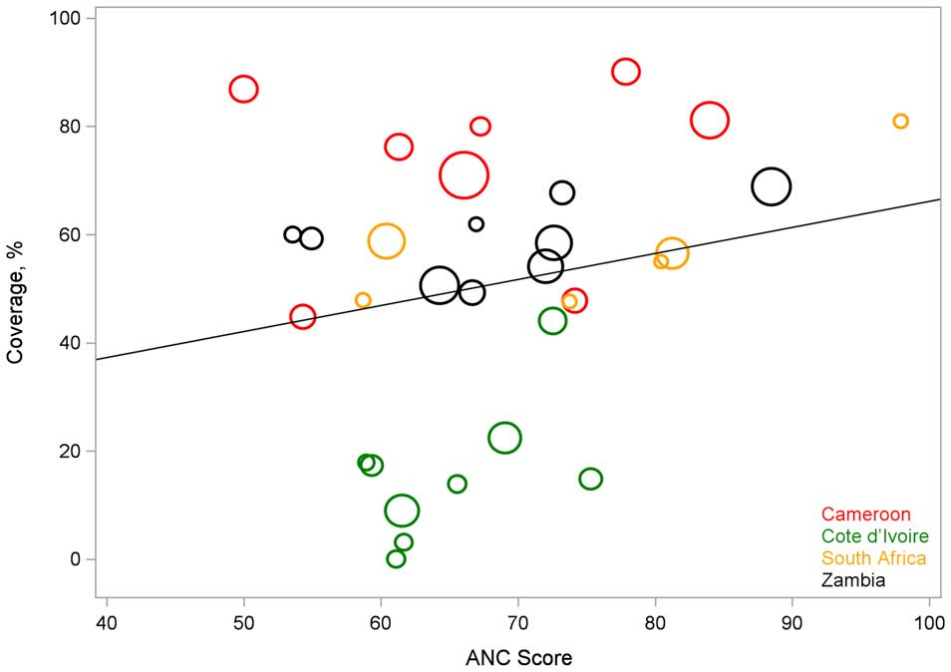
Relationship between composite ANC score and PMTCT coverage. PEARL Facility Survey, 2007–2009.

**Figure 2:**
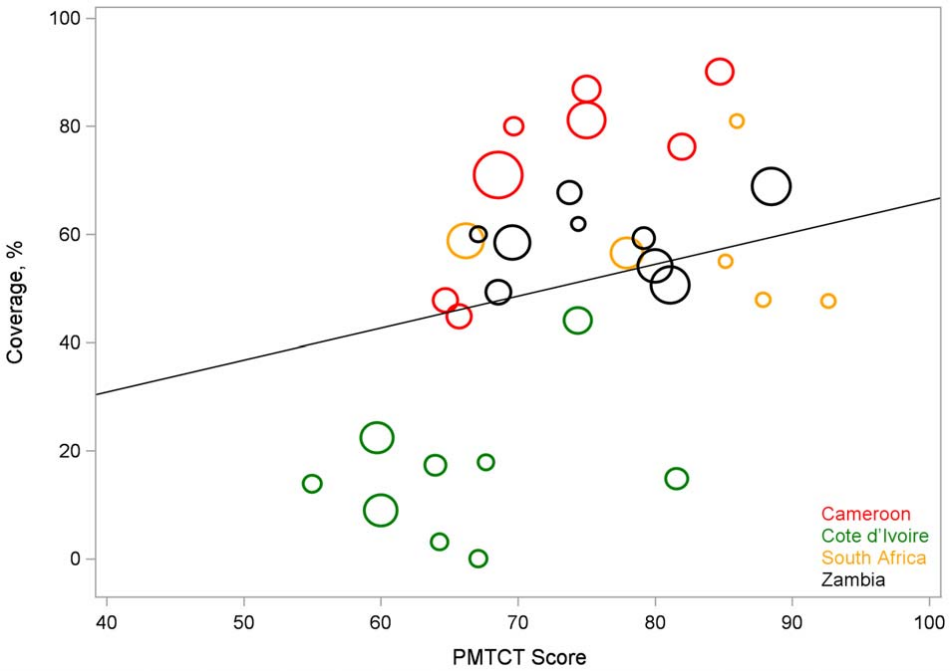
Relationship of composite PMTCT score and PMTCT coverage, PEARL Facility Survey, 2007–2009. Areas of the circles are proportional to the size of the cord blood sample used to estimate PMTCT coverage Line is based on the GEE model that accounts for clustering.

When we accounted for clustering using GEE, only antenatal quality score (p<0.001) remained significantly positively correlated with PMTCT coverage (Figure 1), but PMTCT quality score and patient satisfaction score remained marginally significant (Figure 2).

## Discussion

This facility survey was conducted in sites at which we already knew the PMTCT coverage level from the previously-published cord blood study [5]. The exercise thus presented a unique opportunity to both describe facility factors associated with PMTCT coverage and to begin to validate the survey tool as a predictor of program performance. To our knowledge, this is the first study to examine in a systematic way the relationship between antenatal clinic and service characteristics and an objective outcome such as PMTCT coverage.

Many variables were associated with higher coverage, both individually and in two of the aggregate scores. Some, including availability of CD4 testing and infant PCR testing were intuitively satisfying and agree with our preconceived notions about how programs evolve to be more sophisticated and more successful. We did learn a few new things, however, including that variables pertaining to general antenatal care were even more predictive than PMTCT variables, and that the single factor distinguishing all of the worst-performing sites from the others was the lack of registers with PMTCT information. The predictive value of the non-PMTCT variables, and the lack of predictiveness of several PMTCT variables, reminds us that complex PMTCT services cannot be expected to function well where overall services are poor, even if all the elements of PMTCT are provided.

Overall, we concluded that this tool has limited but not zero utility as a method of evaluating PMTCT sites. Certainly programs do need to know if the essential elements of PMTCT and antenatal care are in place, and common sense dictates that PMTCT requires the presence of drugs, trained staff, and properly stored test kits. However, presence of these elements does not necessarily ensure successful implementation, and answers obtained in this simple checklist-based survey and patient exit interview do not necessarily reflect the experiences of patients visiting the facilities. The overall low coverage of NVP in our study sites (55%, range 33% to 68%) indicates that something is amiss in these PMTCT sites. The aggregate PMTCT score did have some relationship with coverage overall, but not in all countries, and the PMTCT scores were relatively homogeneous compared to the very large observed differences in coverage. Provision of longitudinal medical care and the population's receptiveness to this medical care is a complex process dependent upon attitudes, beliefs, trust, relationships, and complex elements of clinic flow and individual interactions. Most of this cannot be adequately described using simple inventories such as this one, even with direct observation of some elements of care.

Several variables that we expected to be associated with higher PMTCT coverage were not, and on close analysis of the questions and data we appreciated weaknesses in the survey tool that are important to help guide future program evaluation efforts. For example, we asked clinic managers if their site conducted “opt-out” HIV testing. Though several studies have shown that opt-out testing increases uptake [9], [10]. There was no association between opt-out testing and higher PMTCT coverage in our study. Asking clinic managers was probably not the right approach. In an operational sense, opt-out testing is defined not by the policy in place, but by the details of how testing is explained to clients and how they, their laboratory forms, and their blood move through the clinic during their visit. A checklist survey approach does not lend itself to an adequate description of how this occurs, and it is doubtful that we truly distinguished between opt-out and opt-in approaches. There are other examples as well. We found that availability of antiretroviral therapy (ART) at the clinic did not predict PMTCT coverage. Simply having ART at the same site does not tell us how well the ART and antenatal clinics work together to ensure that all eligible women receive ART during pregnancy. The details of *how* the services are integrated will determine successful provision of ART in pregnancy. Meaningful exploration of these issues requires a different type of evaluation.

Our study has several recognized limitations. Since we decided to include only sites that provided both antenatal and delivery services, we included a relatively small number of facilities across four countries. The small sample size may have impacted the statistical power to detect associations between our composite scores as well as individual variables and PMTCT coverage. Second, with only six consecutive observations per facility selected, it is possible that the result for any one site might not be representative of that site's performance (sampling error). This could lead to exposure misclassification and undermine the ability to observe a true association.

Our results broadly support the general principle of strengthening the health care system as an important strategy for improving PMTCT coverage. This analysis exercise provides an important reminder for programs that service provision is complex, and that provision of drugs, test kits, policies, and training does not ensure program success. It is also an important caution to program evaluators that even this carefully conceived and detailed site survey could not predict which sites did well in a very useful way. Once basic elements of PMTCT are in place, detailed clinic-level quality improvement and problem solving initiatives that focus on operational factors are probably just as essential. Further study surrounding quality improvement methods and their impact on coverage and outcomes is warranted.

### Members of the Pearl Study Team

Cameroon: Pius Tih (Cameroon Baptist Health Convention Health Board [CBCHB]); Thomas Welty (CBHC); Allison Spensley (Elizabeth Glaser Pediatric AIDS Foundation [EGPAF]), Christophe Grundman (EGPAF); Catherine Wilfert (EGPAF and Duke University).

Cote d'Ivoire: Didier Koumavi Ekouevi (Programme PAC-CI, Abidjan, Cote d'Ivoire and Université Victor Segalen, Bordeaux, France); Francois Dabis (Université Victor Segalen, Bordeaux, France); Serge Kanhon (Ministry of Health, Abidjan, Cote d'Ivoire).

South Africa: David Coetzee (University of Cape Town, Cape Town, South Africa [UCT]); Kathryn Stinson (UCT); Peter Smith (UCT); Andrew Boulle (UCT); Felicity Gopolang (UCT).

Zambia: Elizabeth M. Stringer; Jeffrey S A Stringer (Centre for Infectious Disease Research in Zambia [CIDRZ]); Benjamin H Chi (CIDRZ), Namwinga Chintu (CIDRZ); Mark Giganti (CIDRZ); Jessica Joseph (CIDRZ), Maximilian Bweupe (Zambian Ministry of Health); Nande Putta (CIDRZ); Alain DeGroot (CIDRZ), Humphrey Mulenga (CIDRZ); Wendy Z Mazimba (CIDRZ); Andrew O. Westfall (CIDRZ), Marc Bulterys (CDC-Zambia); Lawrence H Marum (CDC-Zambia); and Charles Cowan (Analytic Focus, LLC).

US Centers for Disease Control and Prevention: Tracy Creek (National Center for HIV, STD, and TB Prevention, Global AIDS Program, Atlanta Georgia).

World Health Organization: Nathan Shaffer (HIV Department, Geneva; formerly with CDC).

## Supporting Information

Table S1 (XLSX)

Table S2 (XLSX)
